# Lacunas na abordagem da violência sexual contra a mulher: a quem de fato estamos protegendo?

**DOI:** 10.1590/0102-311XPT058424

**Published:** 2024-08-23

**Authors:** Meire Rose de Oliveira Loureiro Cassini, Adalgisa Peixoto Ribeiro, Graziella Lage Oliveira

**Affiliations:** 1 Universidade Federal de Minas Gerais, Belo Horizonte, Brasil.

A violência sexual contra a mulher é um importante problema de saúde pública não apenas por seus impactos à saúde das vítimas e ao sistema de saúde, mas também por ser uma das formas mais graves de violação de direitos humanos, sexuais e reprodutivos ^1^. Os impactos desse tipo de violência atingem de forma contundente as mulheres e de forma indireta toda a sociedade que presencia, no cotidiano, as desigualdades de gênero que subjugam as mulheres e as tornam vulneráveis a problemas de saúde física e mental. Soma-se a isso a sensação de impunidade aos casos de violência sexual que chegam a ser registrados pelo sistema de justiça.

É nesse contexto, que o livro *Violência Sexual contra a Mulher: Abordagens, Contexto e Desafios* ^2^ se coloca, com reflexões a partir da produção de conhecimento em torno da violência de gênero e importantes aspectos relacionados à formação de profissionais, análises epidemiológicas e de ações para seu enfrentamento. Organizado pela pesquisadora Ludmila Fontenele Cavalcanti, líder do Grupo de Pesquisa e Extensão Prevenção da Violência Sexual, da Escola de Serviço Social da Universidade Federal do Rio de Janeiro, o livro está estruturado em três partes, cada uma composta por cinco capítulos. A coletânea agrega pesquisadores de sete instituições de ensino e pesquisa brasileiras e uma colombiana, que se dedicam aos temas relacionados às diferentes expressões da violência de gênero.

A primeira parte do livro aborda a inserção do tema da violência sexual na formação profissional, do ponto de vista dos currículos de graduação em Psicologia, Serviço Social, Medicina e Enfermagem e na perspectiva dos docentes da área da saúde. Apresenta a visão dos profissionais produtores de cuidados às mulheres em situação de violência sexual sobre ações de capacitação, supervisão e suporte institucional. Os autores trazem à tona, ainda, uma discussão sobre a complexidade e as dificuldades enfrentadas para a concretização da interrupção da gestação em casos previstos em lei. Fica evidente, nessa primeira parte do livro, a necessidade de se melhorar a formação dos profissionais de saúde para lidar com a violência contra a mulher, não apenas no aspecto técnico e legislativo, mas também na forma de abordagem e na urgência de um atendimento humanizado às vítimas. Esse é um desafio que ainda persiste na agenda do setor da saúde: capacitar profissionais para lidar com a identificação, abordagem e encaminhamento de casos de violência no Brasil ^3^ ^,^ ^4^.

A segunda parte do livro está voltada aos aspectos conceituais que estariam na gênese da violência contra a mulher. Todos os capítulos inclusos nessa parte abordam de certa forma os mecanismos culturais envolvidos na violência sexual contra a mulher e fazem alusão à estratégia de controle de seus corpos como um “direito” facultado aos homens e ratificado pelo Estado. Mais que uma expressão do poder do homem sobre o corpo feminino, essa parte do livro exemplifica as diversas manifestações do patriarcado ao longo da história, das violências de gênero e da violência institucional contra as mulheres. Questões culturais envolvidas no que os autores chamam de origem da violência de gênero são discutidas a partir da “cultura do estupro”, da violência obstétrica em mulheres negras, do feminicídio como o extremo da violência de gênero praticada contra mulheres, da “pornografia de vingança” que utiliza as mídias digitais causando sofrimento, adoecimento e danos à imagem social e emocional das mulheres, e finalizam com os desafios envolvidos no atendimento a situações de violência sexual contra menores de 14 anos.

Embora os temas do patriarcado e da violência de gênero estejam presentes de forma transversal em todo o livro, é nessa segunda parte que eles são mais enfaticamente discutidos. Esse aspecto é de fundamental importância para o descortinamento das engrenagens envolvidas na violência contra a mulher e dialogam com os aspectos apontados por Foucault ^5^ acerca do papel da violência como um instrumento utilizado para manter a estrutura de poder. No caso, o poder dos homens sobre o corpo das mulheres.

A última parte do livro é dedicada às expressões da violência de gênero contidas nas políticas públicas que abordam a proteção às mulheres. Mais que uma discussão acerca da existência de uma política que contemple o tema, o livro aponta para os desafios na implementação efetiva dessas para a proteção das vítimas de violência sexual. São discutidas a ineficiência do Estado em garantir os direitos das mulheres e a ocorrência de revitimização e perpetuação da violência nas diversas instâncias da rede de proteção, inclusive nos serviços de saúde, nas instituições jurídicas e nas estratégias de promoção e prevenção da violência. Tais aspectos são ilustrados nas seguintes situações: (i) a complexidade acerca da proteção às mulheres que sofreram violência doméstica após a separação de um parceiro violento, cuja violência é mantida após o rompimento do vínculo nas diversas instâncias em que a mulher busca ajuda; (ii) o silêncio existente nas políticas públicas no que tange à prevenção da violência sexual, que não inclui o agressor como ponto central das ações de prevenção; (iii) a violência sexual praticada contra mulheres e crianças em situação de refúgio, mostrando as dificuldades nos processos de proteção social desse grupo; (iv) a descontinuidade das políticas e estratégias de promoção de cuidados às vítimas e de prevenção da violência, que se alteram a cada mudança de governo, dificultando o trabalho em rede intersetorial nos campos da saúde (desde a atenção primária até a atenção especializada); e (v) as fragilidades da atuação em rede, em serviços especializados ou não, e da atuação multidisciplinar e multiprofissional.

O livro chama atenção para o fato de que as violências contra a mulher são decorrentes de misoginia, de discriminação e da desigualdade de poder entre homens e mulheres ao longo da história, ocasionando desfechos desfavoráveis a elas, em muitos casos fatais. Adverte para a necessidade de responsabilização do Estado, tomando para si a formulação de intervenções e ações integradas e intersetoriais para as políticas públicas que de fato garantam os direitos das mulheres, punição a seus agressores e previnam as abordagens desastrosas que as revitimizam.

Os autores encorajam uma atuação que desnaturaliza o desequilíbrio de poder entre homens e mulheres que subjuga a mulher ao lugar de vítima e a culpa por sua condição. Muitos são os exemplos de mulheres sendo julgadas e condenadas moral e socialmente, e revitimizadas pela impunidade de seus agressores. O caso mais recente e ilustrativo disso veio à tona nas mídias sociais por ocasião do julgamento do jogador de futebol brasileiro Daniel Alves, condenado por estupro na Espanha. Apesar de condenado pela justiça, o jogador teve sua liberdade garantida após o pagamento de uma fiança equivalente a R$ 5,4 milhões, enquanto são examinados os recursos contra sua condenação. Em entrevista, a advogada da vítima disse que sua cliente afirmou: “*sinto que voltaram a me estuprar. Estou cansada de ser forte, não quero mais ser forte*” ^6^. Esse fato ilustra a fragilidade do Estado em garantir o direito das mulheres.

No campo da Saúde Coletiva, o livro se faz importante ferramenta de consulta e discussão sobre o tema, apontando caminhos e reconhecendo a violência sexual como uma dimensão da violência de gênero, com caráter interseccional e relacionado às determinações sociais. Traz à tona a importância e a urgência de produzir metodologias de intervenção no atendimento às vítimas e de promover a formação e o acompanhamento dos profissionais que atuam na abordagem das vítimas como fator determinante para a qualidade do cuidado. Embora o livro não sugira como fazê-lo, chama atenção para a necessidade de incluir os agressores em ações que visem à revisão de suas condutas, à prevenção das violências e de proteger e garantir os direitos das mulheres por meio da ação coordenada e integrada das diversas instâncias e políticas públicas estatais.
